# Determinants of HPV vaccine uptake intentions in Chinese clinical interns: an extended theory of planned behavior approach

**DOI:** 10.3389/fpubh.2024.1345530

**Published:** 2024-02-16

**Authors:** Huizi Wang, Yuedong Xu, Hui Zhang, Ning Chen

**Affiliations:** ^1^Department of Nursing, The First Affiliated Hospital of Shandong First Medical University & Shandong Provincial QianFoshan Hospital, Jinan, China; ^2^Center of Digestive Endoscopy, Shandong Provincial Hospital Affiliated to Shandong First Medical University, Jinan, China

**Keywords:** HPV vaccine, clinical interns, theory of planned behavior (TPB), health knowledge, vaccination intention

## Abstract

**Background:**

This study aims to utilize the extended Theory of Planned Behavior (TPB) model to examine the intentions of clinical interns in China towards Human papillomaviruses (HPV) vaccination. It also fills a significant gap in the literature concerning vaccine acceptance in this specific population.

**Methods:**

This cross-sectional study was carried out with clinical interns in Shandong Province, China, with a total of 1,619 participants. Data were collected through self-reported questionnaires, including demographic characteristics, TPB variables, and HPV-related health knowledge. Hierarchical regression analysis was employed to identify key factors influencing vaccination intentions, and Structural Equation Modeling (SEM) was used to analyze the interrelationships between these factors.

**Results:**

This study initially identified key predictors affecting clinical interns’ intentions to receive the HPV vaccine through hierarchical regression analysis. The preliminary model, which accounted for demographic factors, revealed foundational impacts of household income and HPV-related clinical experience on intentions. After integrating TPB variables—attitude, subjective norm, perceived behavioral control, and HPV-related health knowledge—the model’s explanatory power was enhanced to 37.30%. SEM analysis focused on the interplay among TPB constructs and extended variables, confirming their significance in forming vaccination intentions, with subjective norm having the most substantial impact (β = 0.375, *p* < 0.001). The extended TPB model explained over half of the variance in vaccination intentions, substantiating the hypotheses and revealing the psychological determinants behind clinical interns’ decision-making for HPV vaccination.

**Conclusion:**

The extended TPB model from this study effectively explains the vaccination intentions among clinical interns for HPV, offering theoretical support for public health strategies and educational interventions targeting this group. These findings are of significant importance for public health practice and future health promotion strategies.

## 1 Introduction

Human papillomaviruses (HPV), a diverse group of small DNA viruses that primarily target the mucosal and epidermal squamous epithelium, leading to benign warts and malignant tumors in the anogenital region and upper respiratory and digestive tracts (1). Specifically, high-risk HPV subtypes are instrumental in the development of various pathologies, including oropharyngeal malignancies, cervical cancer, male genital tumors, and dermatological conditions like condyloma acuminatum (2). Contemporary epidemiological assessments posit that roughly 2.8 billion women aged 15 years and onward are at potential risk for cervical cancer initiation (3). In China, with its substantial population, there are approximately 106,000 new cases of cervical cancer and 48,000 fatalities annually, constituting over 28% of the worldwide cases of this disease (4). Furthermore, the incidence of HPV-associated head and neck cancers is escalating swiftly across China, and it is projected that HPV-positive oropharyngeal cancer cases will exceed cervical cancer cases within the next 15–20 years (2).

Nonetheless, for a set of malignancies with a well-established etiology (5), comprehensive clinical investigations have underscored that prophylactic vaccination before initial sexual exposure ensures efficacy against the designated HPV strains (6). Subsequent to the 2006 endorsement of the HPV vaccine in the United States, over 100 nations have formally adopted it into their respective national immunization schedules (7). Unfortunately, due to public concerns over the safety and risk of harm from vaccinations, vaccine coverage rates are slowly declining in developed nations (8). In alignment with the “Accelerated Eradication of Cervical Cancer” initiative advanced by the World Health Organization (WHO), several cities within mainland China have commenced pilot HPV immunization programs for adolescent girls from 2020 onwards (9). Similarly, due to the lack of vaccine accessibility for adult females and parental concerns regarding the safety of vaccines, the current HPV vaccination rate in China is considerably low, at less than 6% of the overall population (10). In the landscape of HPV vaccination, clinical interns represent a distinctive cohort, encompassing the forthcoming cadre of physicians, nurses, pharmacists, and other primary healthcare professionals. They are at the forefront of advancing public health responsibilities, playing a crucial role in the promotion and popularization of HPV vaccination (2). Numerous studies have demonstrated that healthcare professionals are the primary providers of vaccinations, and individuals are more likely to get vaccinated following their recommendation (11, 12). Notably, clinical interns are also within the age group most at risk for HPV infection (13). As the HPV vaccine becomes increasingly available in China, understanding and elevating the vaccine uptake intentions among this demographic is critical. Despite the increasing spread of HPV vaccination, a noticeable gap remains in the literature regarding the vaccine’s acceptance among Chinese clinical interns. Consequently, this study seeks to evaluate the vaccination intentions of Chinese clinical interns and to identify the critical factors influencing these intentions.

Earlier research has identified a range of factors influencing HPV vaccine acceptability in the general population, including knowledge about the vaccine, perceptions of its safety, and accessibility to vaccination (2, 12, 13). However, for this specific cohort, relying solely on descriptive research methodologies may not sufficiently probe the underlying reasons for vaccine acceptability. The Theory of Planned Behavior (TPB) is a well-established model in social psychology, delineating the relationship between individuals’ intention and their resultant behaviors (14). It postulates that an individual’s behavior is influenced by their intention, which is shaped by attitude towards the behavior, subjective norms, and perceived control over the action (15). Previous research findings have indicated that within the TPB framework, attitudes, subjective norms, and perceived control are positively associated with behavioral intentions (3, 16). Furthermore, TPB is adaptable, and Ajzen suggests that it can be adapted to incorporate additional variables (15). In the expanded framework of the TPB, additional psychosocial constructs such as media engagement, HPV risk and vaccination perceptions, descriptive norms, and anticipated regrets have been incorporated to assess their influence on the intent to get the HPV vaccine (4, 14, 15). Moreover, Rajeh et al. (16) augmented the TPB model by adding perceived knowledge as a predictive variable for oral health behavior intentions. Beyond the core components of the TPB model, health knowledge related to HPV will serve as potential determinants influencing vaccination intentions in this study. As a result, this study includes perceptual knowledge into the model and employs Structural Equation Modeling (SEM) to analyze the effectiveness of the extended TPB model in explaining clinical interns’ intentions to receive the HPV vaccine, thereby providing a more comprehensive theoretical support and predictive capability.

## 2 Materials and methods

### 2.1 Study design and participants

This study conducted from September to November 2023 aimed to investigate the prevalence and perceptions of HPV vaccination among medical interns in Shandong Province, a densely populated region in eastern China. A convenience sampling strategy was employed, targeting clinical interns slated for the 2023–2024 academic year. Data collection was collected using Wenjuanxing, a widely used Chinese online survey platform. To maximize survey reach, an invitation link was distributed through WeChat app—a prominent social networking platform in China—specifically within intern group chats across various hospitals. In an effort to bolster the study’s credibility and minimize duplicate responses, participants were mandated to authenticate using their WeChat login credentials prior to survey completion. Furthermore, to mitigate hasty or insincere responses, a minimum completion time of 5 min was enforced. All participants were informed about the study’s aims and gave informed consent. Ethical approval for the research was granted by the Ethics Committee at the First Affiliated Hospital of Shandong First Medical University & Shandong Provincial QianFoshan Hospital (NO. S467).

#### 2.1.1 Theoretical framework and hypothesis development

The theoretical underpinning of this study is anchored in the extended TPB model, which posits that an individual’s intention to perform a behavior is influenced by attitudes towards the behavior, subjective norms, and perceived behavioral control. In addition, we have also included health knowledge related to HPV (HPVK) as an independent variable, as evidence suggests that knowledge can exert a significant direct influence on health behaviors within an extended TPB model (17). Therefore, we propose:

*H1a:* The attitudes of clinical interns towards HPV vaccination positively correlate with their willingness to be vaccinated.

*H2a:* There is a positive relationship between subjective norm and clinical interns’ inclination to receive the HPV vaccination.

*H3a:* There was a positive correlation between PBC and the willingness of clinical interns to receive the HPV vaccine.

*H4a:* The health knowledge of clinical interns towards HPV vaccination positively correlated with their willingness to be vaccinated.

**Figure 1:**
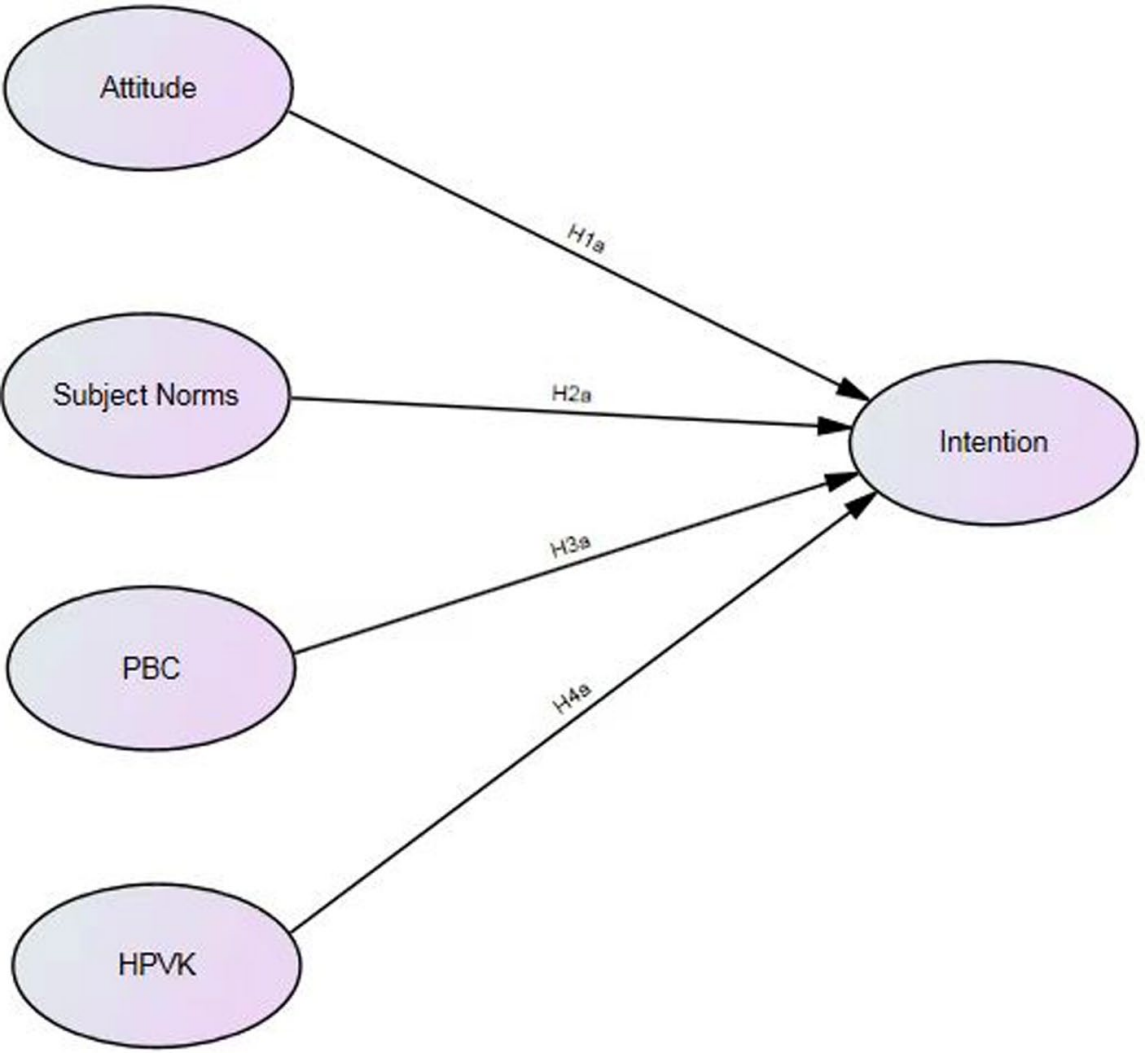
Proposed extended TPB model.

To ensure clarity in the relationships between our constructs, we employed SEM as our analytical strategy. SEM offers a robust framework for examining the direct and indirect relationships between multiple predictors and an outcome variable, while also accounting for measurement error (18). This method is particularly suited for our study’s aims, given the complexity of the relationships we are investigating and the need to understand the independent effects of each variable within the extended TPB framework. Consequently, based on the aforementioned assumptions, an extended TPB model has been developed, as illustrates in Figure 1.

### 2.2 Measures

#### 2.2.1 Socio-demographic questionnaire

The survey instrument was carefully designed to collect essential socio-demographic information, encompassing gender, age, educational background, household monthly income, field of study, relationship status, and HPV related clinical experience.

#### 2.2.2 Attitude

Attitude is defined as an individual’s favorable or unfavorable assessment of a particular behavior (15). The Attitude Scale, adapted from Catalano et al. (19), comprises three items intended to gauge the positive or neutral perspectives of clinical interns regarding HPV vaccination. It includes items such as (a) I think HPV vaccination is very beneficial to my health; (b) I think HPV vaccination is effective in preventing HPV-related diseases; and (c) I think the benefits of HPV vaccination may outweigh the side effects. Participants completed items on a 5-point Likert scale, which spanned from (1) strongly disagree to (5) strongly agree. These items demonstrated high reliability with a Cronbach’s alpha of 0.778.

#### 2.2.3 Subjective norm

The subjective norm is defined as an individual’s belief regarding the extent of support or opposition from important others for performing a specific behavior (15). The Subjective Norms Scale, taken from shah et al. (20), consists of five questions, including: “Would I get the HPV vaccine if recommended by social media, family, friends, a known vaccine recipient, the National Health Commission of China, or a trainee hospital.” Participants finished responses on a 5-point Likert scale ranging from (1) strongly disagree to (5) strongly agree.

The scale demonstrated strong internal consistency (Cronbach’s alpha = 0 0.838).

#### 2.2.4 Perceived behavioral control

Perceived Behavioral Control (PBC) refers to an individual’s belief in their ability to execute a specific behavior or action (15). The PBC Scale, also adapted from Shah et al. (20), emphasizes the confidence of clinical interns in their ability to make an autonomous decision to receive the HPV vaccine. The scale comprises three items, including: “(a) I think it is convenient to get HPV vaccination in my region; (b) I think it is easy for me to get information about HPV vaccination; (c) I think the cost of HPV vaccination is affordable.” Participants provided responses using a 5-point Likert scale, where (1) indicates strong disagreement and (5) indicates strong agreement. The items demonstrated strong internal consistency (Cronbach’s alpha = 0 0.827).

#### 2.2.5 Intention

Intention refers to the readiness of clinical interns to receive the HPV vaccine within the upcoming 6 months. This construct was measured with two 5-point Likert scale items, modified from Yi et al. (3). One representative item stated: “I intend to complete all three doses of the HPV vaccine in the forthcoming 6 months” (Cronbach’s alpha = 0 0.780).

#### 2.2.6 Health knowledge related to HPV

Research indicates a positive connection between an individual’s knowledge concerning HPV and their vaccination status, even within the demographic of medical students (15, 16).

Nonetheless, academic studies have yet to explore the link connecting HPV-related knowledge (HPVK) with vaccination intentions among clinical interns in China. Within this research, the HPV-related health literacy knowledge of clinical interns was assessed using six items derived from Shah et al. (20)(Cronbach’s alpha = 0 0.883). These items cover essential information on HPV, vaccination importance, and related health risks, which might significantly impact clinical interns’ decision-making. (Details are provided in the Supplementary materials).

### 2.3 Data analysis

This study employed a two-stage statistical approach to test the research hypotheses. Initially, descriptive statistics and frequency distributions were generated using SPSS version 26.0 to characterize the sociodemographic attributes of the study participants. Subsequently, Hierarchical Linear Regression analysis was carried out in three steps to investigate the impact of the predictor variables on the intention. The first step accounted for demographic factors to control for basic individual differences. The second step introduced the core components of the TPB—attitude, subjective norm, and PBC. The third step added additional TPB-related variables, including knowledge, to assess their incremental impact on the model. This hierarchical approach not only revealed the unique contribution of each set of variables in predicting intention after controlling for others but also allowed us to evaluate the incremental explanatory power of the newly added variables.

Further, SEM analysis was executed in IBM SPSS Amos 26.0 to collectively test the research hypotheses. The SEM analysis took into account the interactions between all variables, assessing not only the direct impact of attitude, subjective norm, PBC, and knowledge on intention but also the overall fit of the theoretical model. Prior to conducting Confirmatory Factor Analysis (CFA), the measurement model was subjected to an assessment of construct cohesiveness using Cronbach’s alpha to ensure the constructs’ dependability. Subsequently, CFA was employed to confirm the reliability and validity of each variable. Average Variance Extracted (AVE) and Composite Reliability (CR) were used to evaluate convergent validity. To affirm the analytical results’ precision, the study employed various fit metrics: Root Mean Square Error of Approximation (RMSEA), Comparative Fit Index (CFI), Goodness of Fit Index (GFI), and the Adjusted Goodness of Fit Index (AGFI).

## 3 Results

### 3.1 Demographic characteristics of participants

From September to November 2023, a total of 1,619 responses were collected. The characteristics of the respondents are summarized in Table 1. The majority of respondents were predominantly within the age range of 20–22 years (*n* = 873, 53.8%), and more than two-thirds of the participants were female (*n* = 1,238, 70.2%). Additionally, almost all respondents possessed at least a college degree (*n* = 1,586, 98%). Pharmacy (52.7%) and nursing (31.3%) emerged as the most fields of study. A significant number of the respondents (74.2%) reported a monthly household income below RMB 8,000 ($1,100 USD). It is noteworthy that 34.5% of the respondents reported having dealt with HPV-related diseases during their clinical internships.

**Table 1:** Demographic characteristics of participants.

Characteristics		Frequency (%)
Gender	Male	336 (20%)
	Female	1,283 (79.2%)
Age (years)	<20	708 (43.7%)
	20–22	871 (53.8%)
	23–25	29 (1.8%)
	>25	11 (0.7%)
Education background	High school or less	33 (2%)
	College	1,376 (85%)
	Bachelor’s degree or higher	210 (13%)
Monthly household income (CNY)	<5,000	634 (39.2%)
	5,000–8,000	566 (35%)
	8,000–13,000	291 (18%)
	>13,000	128 (8%)
Major	Clinical medicine	70 (4.3%)
	Nursing	506 (31.3%)
	Pharmacy	853 (52.7%)
	Public health	190 (11.8%)
Relationship status	Married	18 (1.1%)
	In a relationship	301 (18.6%)
	Single	1,300 (80.3%)
HPV clinical experience	Yes	558 (34.5%)
	No	1,061 (65.5%)

### 3.2 Hierarchical regression analysis

Hierarchical regression analysis was utilized to determine the influence of demographic factors, TPB components, and HPV-related health knowledge on the intentions to receive HPV vaccination (refer to Table 2 for detailed statistics). Model 1 incorporated demographic factors, revealing that monthly household income was a significant predictor of vaccination intention. Specifically, interns hailing from households with an income exceeding RMB 8,000 ($1,100 USD) per month were more inclined towards vaccination compared to their counterparts from households earning below RMB 5,000 ($690USD) monthly (β = 0.091; *p* < 0.01). Furthermore, having HPV-related clinical experience also emerged as a positive determinant (β = 0.114; *p* < 0.001). Other demographic variables such as age, gender, marital status, educational attainment, and field of study did not present significant effects.

**Table 2:** Hierarchical multiple regression analysis.

Variables	Model 1		Model 2		Model 3	
	B(SE)	β	B(SE)	β	B(SE)	β
Constant	3.810 (0.286) ^***^		0.444 (0.272) ^***^		0.415 (0.263) ^**^	
Age	0.077 (0.044)	0.047	0.015 (0.037)	0.009	−0.035 (0.036)	−0.029
Gender	−0.047 (0.056)	−0.021	−0.053 (0.047)	−0.023	−0.065 (0.045)	−0.021
Education background	0.080 (0.022)	−0.003	0.021 (0.053)	0.390	−0.013 (0.051)	−0.006
Monthly household income	0.080 (0.022) ^**^	0.091	0.042 (0.018) ^**^	0.047	0.040 (0.018) ^**^	0.045
Major	−0.021 (0.024)	−0.022	0.012 (0.020)	0.013	0.024 (0.019)	0.025
Relationship Status	−0.056 (0.053)	−0.027	−0.031 (0.044)	−0.015	−0.039 (0.043)	−0.018
HPV health education	0.220 (0.048) ^***^	0.114	0.076 (0.041) ^**^	0.040	0.056 (0.039) ^**^	0.029
Attitude			0.151 (0.026) ^***^	0.131	0.143 (0.025) ^***^	0.124
Subject norms			0.449 (0.030) ^***^	0.351	0.403 (0.029) ^***^	0.315
Perceived behavioral control			0.219 (0.021) ^***^	0.238	0.226 (0.020) ^***^	0.245
Health knowledge related to HPV					0.123 (0.011) ^***^	0.221
Δ*R*^2^	0.026^***^		0.324^***^		0.369^***^	
*F* value	7.104^***^		231.648^***^		115.380^***^	

* *p* < 0.05; ** *p* < 0.01; *** *p* < 0.001. B, standardized regression coefficient; SE, standard error of standardized regression coefficient; β, unstandardized regression coefficient.

In Model 2, the incorporation of TPB constructs—attitude, perceived behavioral control, and subjective norms—led to a significant enhancement in the model’s explanatory power. This inclusion increased the variance explained to 32.3% (*F* = 231.648, *p* < 0.001), highlighting the substantial influence of these psychosocial factors.

Extending the analysis to Model 3 further confirmed the association between TPB and its extensions and vaccination intentions. Notably, subjective norms held the most substantial weight (β = 0.315, *p* < 0.001), followed by perceived behavioral control (β = 0.245, *p* < 0.001), knowledge (β = 0.221, *p* < 0.001), and attitude (β = 0.124, *p* < 0.001). The final model accounted for 37.30% of the variance in vaccination intentions (*F* = 115.380, *p* < 0.001), signifying the multifaceted nature of the decision-making process related to HPV vaccination.

### 3.3 Confirmatory factor analysis

Our analysis began with a detailed examination of the factor loadings for each item within the scale. Guided by established criteria (21), items with loadings below the critical threshold of 0.5 were omitted to refine the measurement model and enhance construct validity (HPVK20). Subsequently, an extensive reliability and validity assessment was performed on the revised scale, with results presented in Table 3, confirming that each item met satisfactory levels of reliability and validity. Additionally, the overall model fit was meticulously evaluated, yielding favorable indices: GFI = 0.976, CFI = 0.981, AGFI = 0.967, RMSEA = 0.032, indicating a high degree of fit with the empirical data. Lastly, the study highlighted the discriminant capacity of our research model by ensuring that the square root of each factor’s AVE exceeded the corresponding correlation coefficients among constructs (22) (see Table 4).

**Table 3:** Convergent and reliability validity of the revised scale.

Construct	Item	Standardized loadings	AVE	CR	Reliability coefficient (α)
Attitude	AT1	0.74	0.542	0.780	0.778
	AT2	0.75			
	AT3	0.70			
Subject Norms	SN1	0.732	0.511	0.839	0.838
	SN2	0.695			
	SN3	0.743			
	SN4	0.688			
	SN5	0.714			
Perceived behavioral control	PBC 1	0.824	0.618	0.829	0.827
	PBC2	0.748			
	PBC 3	0.784			
Health knowledge related to HPV	HPVK15	0.761	0.838	0.512	0.833
	HPVK 16	0.731			
	HPVK 17	0.635			
	HPVK 18	0.821			
	HPVK 19	0.607			
Intention	IN1	0.827	0.788	0.650	0.780
	IN2	0.785			

**Table 4:** Discriminant validity of the revised scale.

Construct	Attitude	Subject norms	PBC	HPVK	Intention
Attitude	0.542				
Subject norms	0.382***	0.511			
PBC	0.263***	0.369***	0.618		
HPVK	0.107***	0.186***	0.052*	0.512	
Intention	0.332***	0.498***	0.411***	0.303***	0.650

* *p* < 0.05; ** *p* < 0.01; *** *p* < 0.001.

### 3.4 Structural equation model

The extended TPB-based model was analyzed using the Structural Equation Modeling (SEM) (Table 5). The fit indices demonstrated a strong alignment of the extended TPB model with the dataset, as shown by a GFI of 0.978, CFI of 0.981, AGFI of 0.967, and RMSEA of 0.032. The path coefficient analysis revealed that attitude (β = 0.119, *p* < 0.001), subjective norms (β = 0.375, *p* = *p* < 0.001), and perceived behavioral control (β = 0.289, *p* < 0.001), along with significant HPV health knowledge (β = 0.248, *p* < 0.001), positively influenced intentions to vaccinate. Accordingly, hypotheses H1a, H2a, H3a, and H4a are supported within the extended TPB structural model. The model accounted for 51.5% of the variance in intentions to vaccinate.

**Table 5:** Structural equation model of the study hypotheses.

	Beta- coefficient	SE	*p*
Intention<−-Attitude	0.119	0.038	***
Intention<−-Subject Norms	0.375	0.043	***
Intention<−-Perceived Behavioral Control	0.289	0.029	***
Intention<−-Health Knowledge related to HPV	0.248	0.071	***

*** *p* < 0.001.

## 4 Discussion

This study extensively investigates the determinants influencing HPV vaccine uptake intentions among clinical interns in China, utilizing elements of the TPB model and extended factors such as HPV-related health knowledge. Our findings emphasize the significant roles of TPB’s core components—attitudes, subjective norms, and perceived behavioral control—in forming vaccination intentions. Concurrently, HPV-related health knowledge, as a vital independent variable, plays a crucial role in influencing the vaccination decisions of clinical interns. Additionally, the study examines the potential impact of demographic factors on vaccination intentions. These insights provide important perspectives for understanding and promoting HPV vaccination among future healthcare professionals.

The research indicated that subjective norms are the most significant determinant of clinical interns’ intentions to vaccinate against HPV, corroborating earlier TPB-based studies on vaccination intent predictions (20, 23, 24). Within the TPB framework, subjective norms refer to an individual’s perception of how significant others view their behavior, reflecting the importance of social approval in decision-making (15). For clinical interns, this suggests they may particularly value the opinions of peers, mentors, and authoritative health organizations. Their perspectives not only reflect societal endorsement but also represent the professional standards and expectations they aspire to as newcomers to the medical profession. In this context, clinical interns’ perceptions of what is socially approved are influenced by the information they access, making it evident that the quality and accessibility of information significantly contribute to their vaccination decisions (25). Reinforcing positive attitudes and information about the HPV vaccine in medical education and professional training can enhance interns’ willingness to vaccinate as they build their professional identities (25).

Additionally, our study observed a significant positive correlation between Perceived Behavioral Control (PBC) and the intention to receive the HPV vaccine, aligning with the expectation that higher perceived self-efficacy and stronger subjective norms typically enhance the intention to engage in specific behaviors (15). However, this finding contrasts with studies from certain regions in the United States. For instance, a study conducted within the framework of the TPB in Alabama found no correlation between PBC and university students’ intentions to vaccinate HPV (20). Similarly, a study in Arizona reported that immunizing pharmacists and pharmacy interns scored lower on perceived subjective norms compared to other TPB constructs (23). This discrepancy is primarily reflected in the United States’ introduction of the HPV vaccine for females in 2006 and the implementation of a gender-inclusive HPV immunization policy in 2011 (26), significantly enhancing vaccine uptake and economic accessibility. This policy provides the general population, especially adolescents and young adults, with low-cost or free vaccination channels through public health projects or health insurance. Such a strategy effectively reduces the barriers to vaccine administration, potentially diminishing the influence of PBC on the intention to vaccinate. Conversely, in China, despite increasing public awareness of the HPV vaccine, its non-inclusion in the national immunization schedule and the necessity for out-of-pocket payment (3), along with limited vaccination sites, may heighten the barriers related to cost and accessibility. In order to make HPV vaccination more accessible and affordable for a wider population, collaboration and communication among relevant stakeholders are indispensable (25). This collaboration should involve healthcare institutions, government agencies, and public health organizations. Their collective efforts, including the implementation of policies aimed at offering more extensive vaccination support and effectively addressing obstacles to vaccine acceptance, will play a pivotal role in expanding vaccination centers and mitigating the economic challenges associated with vaccination. Among the TPB variables, attitude was identified as the weakest predictor of the intention to receive the HPV vaccine, indicating that the interns’ intentions are less influenced by their personal evaluations and feelings towards the vaccine. This phenomenon could stem from the fact that as novices in the medical profession, their decision-making is more driven by professional standards and medical education guidance (2), rather than merely personal emotional preferences.

Prior research from various nations has consistently revealed that groups with more comprehensive knowledge about HPV, including students of different ages (27, 28), parents of adolescents (29, 30), and men and women from various age groups (31, 32), tend to show a stronger inclination towards vaccination. Despite this, there has been a scarcity of studies exploring the link between these two factors within the TPB framework. To address this gap, our study incorporated “knowledge” as an additional variable in the TPB model to investigate its influence on clinical interns’ intentions to receive the HPV vaccine. The findings indicated a positive correlation between the interns’ intentions to get vaccinated and their understanding of HPV health issues. This implies that clinical interns, who have acquired a more in-depth comprehension of HPV and its vaccine through their medical education (13), are likely to give more weight to preventive measures. Moreover, their direct involvement in patient care enhances their focus on disease prevention, which in turn further solidifies the transition from knowledge to the intention to vaccinate. In light of these findings, it is recommended to further enhance public health and preventive medicine curricula, with a particular focus on comprehensive training about HPV and its vaccines (33, 34). Teaching hospitals should organize targeted health promotion activities, such as workshops and lectures, aimed at increasing medical trainees’ awareness of the importance of HPV vaccination. Korkidi et al. (35) also underscored the crucial role of health education in the effective execution of HPV vaccine strategies, particularly in enhancing societal awareness and consciousness of HPV prevention.

This research notably shows that specific demographic attributes significantly influence the inclination towards HPV vaccination. Specifically, clinical interns with higher family monthly income and those who have been involved in handling HPV-related cases exhibited a stronger intention to receive the HPV vaccine. In China, due to the specifics of medical education and training programs, intern medical students are typically not considered official employees of their respective hospitals, and they usually do not receive a salary (36), leading to a lack of financial independence. Additionally, the cost of HPV vaccination in China is relatively high, with prices for imported vaccines ranging from approximately RMB 1,800 ($270 USD) to RMB 3,900 ($580 USD), while domestic vaccines are priced at about RMB 1,000 ($150 USD) (37). In this study, the majority of the interns’ family monthly incomes were below RMB 8,000 ($1,100 USD), making the cost of vaccination a significant economic consideration. Consequently, it was observed that interns from families with higher monthly incomes demonstrated a markedly stronger intention to get vaccinated against HPV, clearly highlighting the close relationship between family economic status and vaccination intentions. Additionally, we also observed that clinical interns with experience in handling cases related to HPV exhibited a stronger inclination towards HPV vaccination. This trend may be attributed to the direct involvement of medical practitioners in managing HPV-related cases during their clinical practice (38), leading to a deeper understanding of HPV’s infection pathways and its potential health threats. Such firsthand experience enhanced their knowledge of the efficacy of HPV vaccination, resulting in a higher willingness to opt for vaccination compared to their peers who had not directly handled similar cases. Therefore, during the internship period, it is recommended that teaching hospitals prioritize placing interns in departments that handle HPV cases, enabling students to better comprehend the infection pathways of HPV and its impact on health. While we found a clear link between family economic status and HPV vaccination intentions, as well as a direct correlation with clinical experience with HPV, it is notable that other demographic variables such as gender did not present significant associations in our analysis. The findings stand in contrast to previous reports by Chen et al. (39), which highlighted gender-based differences in HPV vaccination willingness, and López et al. (40), which identified female gender as a significant determinant of vaccine acceptability. This may be indicative of the clinical intern group’s unique characteristics, where their professional identity and practical medical exposure could overshadow the influence of traditional demographic factors. It underscores the need for future studies to explore the multifaceted influences on health behavior intentions within varied demographic contexts.

Our study also has some limitations. Firstly, the study’s exclusive focus on the Shandong region impacts the diversity of its participants. Considering the significant variations in educational resources, medical practices, economic conditions, and cultural norms across different regions of China, the findings might not fully represent the actual situation in other areas of the country. Moreover, only a small proportion of respondents (0.7%) were over the age of 25. This demographic homogeneity limits the generalizability of our findings across a broader age range and to individuals outside the medical industry. Consequently, the specialized knowledge of our respondents about vaccines may not be representative of the general population’s awareness or attitudes towards HPV vaccination. Future research could build upon this by extending its scope to include a more diverse array of participants, thus enabling comparative studies across a broader spectrum of perspectives. Secondly, the cross-sectional design of the study, while useful for identifying correlations between variables, is limited in establishing causality. Additionally, the use of self-reported data might lead to biases like social desirability or recall bias, potentially impacting the precision of the findings. Lastly, while the expanded TPB model offers valuable insights, it lacks integration with components from other behavior change theories, such as the Transtheoretical Model and the Health Belief Model. Future research should consider integrating elements from these and other behavioral theories to enrich the understanding of HPV vaccination intentions among clinical interns.

## 5 Conclusion

This study is among the few worldwide to focuses on the HPV vaccine uptake intentions among clinical interns, representing a pioneering exploration in this field within China. By utilizing the extended theory of planned behavior model, this research highlights the pivotal roles of attitudes, subjective norms, perceived behavioral control, and HPV-related health knowledge in shaping vaccine intentions. Considering the crucial role of future medical professionals in promoting public health and vaccine advocacy, the insights from this study are essential for national authorities in formulating vaccination policies and play a significant role in guiding medical education and training in teaching hospitals.

## Data availability statement

The raw data supporting the conclusions of this article will be made available by the authors, without undue reservation.

## Ethics statement

The studies involving humans were approved by The First Affiliated Hospital of Shandong First Medical University & Shandong Provincial QianFoshan Hospital. The studies were conducted in accordance with the local legislation and institutional requirements. Written informed consent for participation in this study was provided by the participants' legal guardians/next of kin. Written informed consent was obtained from the individual(s) for the publication of any potentially identifiable images or data included in this article.

## Author contributions

HW: Writing – original draft. YX: Writing – review & editing, Data curation, Formal analysis, Project administration. HZ: Supervision, Writing – review & editing. NC: Data curation, Writing – review & editing.
