# Supplementary material for: Determinants of HPV vaccine uptake intentions in Chinese clinical interns: an extended theory of planned behavior approach
Source: Front Public Health. 2024 Feb 16;12:1345530. doi: 10.3389/fpubh.2024.1345530 (PMC10904661; doi:10.3389/fpubh.2024.1345530)
Supplement: Supplementary file 1 [file Table_1.docx]

| Question number | Correct answer |
| --- | --- |
| Q1: Do you think human papillomavirus (HPV) is contagious? | Yes |
| Q2: In which gender can human papillomavirus cause health problems? | In both women and men |
| Q3:What are modes of transmission of HPV? | Sexual intercourse or from mother to baby during birth |
| Q4:Can HPV cause cancer? | Yes |
| Q5:Can HPV cause genital warts? | Yes |
| Q6:How many HPV vaccines are currently available in China? | Five |

Supplementary Material
